# Identification of Polyphenolic Compounds Responsible for Antioxidant, Anti-*Candida* Activities and Nutritional Properties in Different Pistachio (*Pistacia vera* L.) Hull Cultivars

**DOI:** 10.3390/molecules28124772

**Published:** 2023-06-14

**Authors:** Shima Gharibi, Adam Matkowski, Danial Sarfaraz, Hossein Mirhendi, Hamed Fakhim, Antoni Szumny, Mehdi Rahimmalek

**Affiliations:** 1 Core Research Facilities (CRF), Isfahan University of Medical Sciences, Isfahan 81746-73461, Iran; s.gharibi@mail.mui.ac.ir; 2 Department of Pharmaceutical Biology and Botany, Wroclaw Medical University, Borowska 211, 50-556 Wroclaw, Poland; pharmaceutical.biology@wp.eu; 3 Department of Plant Breeding, Isfahan University of Technology, Isfahan 84156-83111, Iran; danial_1372_n@yahoo.com; 4 Department of Medical Parasitology and Mycology, School of Medicine, Isfahan University of Medical Sciences, Isfahan 81746-73461, Iran; s.h.mirhendi@gmail.com; 5 Mycology Reference Laboratory, Research Core Facilities Laboratory, Isfahan University of Medical Sciences, Isfahan 81746-73461, Iran; 6 Infectious Diseases and Tropical Medicine Research Center, Isfahan University of Medical Sciences, Isfahan 81746-73461, Iran; 7 Department of Food Chemistry and Biocatalysis, Wrocław University of Environmental and Life Sciences, 50-375 Wroclaw, Poland; antoni.szumny@upwr.edu.pl (A.S.); mrahimmalek@iut.ac.ir (M.R.); 8 Department of Horticulture, College of Agriculture, Isfahan University of Technology, Isfahan 84156-83111, Iran

**Keywords:** pistachio, antiglycative, phenol, flavonoid, HPLC, antifungal, oil

## Abstract

The use of by-products from the agri-food industry is a promising approach for production of value-added, polyphenol-rich dietary supplements or natural pharmaceutical preparations. During pistachio nut processing, a great amount of husk is removed, leaving large biomass for potential re-use. The present study compares antiglycative, antioxidant, and antifungal activities as well as nutritional values of 12 genotypes belonging to four pistachio cultivars. Antioxidant activity was measured using DPPH and ABTS assays. Antiglycative activity was evaluated as inhibition of advanced glycation end product (AGE) formation in the bovine serum albumin/methylglyoxal model. HPLC analysis was performed to determine the major phenolic compounds. Cyanidin-3-*O*-galactoside (120.81–181.94 mg/100 g DW), gallic acid (27.89–45.25), catechin (7.2–11.01), and eriodictyol-7-*O*-glucoside (7.23–16.02) were the major components. Among genotypes, the highest total flavonol content (14.8 mg quercetin equivalents/g DW) and total phenolic content (262 mg tannic acid equivalent/g DW) were in KAL1 (Kaleghouchi) and FAN2 (Fandoghi), respectively. The highest antioxidant (EC_50_ = 375 μg/mL) and anti-glycative activities were obtained for Fan1. Furthermore, potent inhibitory activity against *Candida* species was recorded with MIC values of 3.12–12.5 µg/mL. The oil content ranged from 5.4% in Fan2 to 7.6% in Akb1. The nutritional parameters of the tested cultivars were highly variable: crude protein (9.8–15.8%), ADF (acid detergent fiber 11.9–18.2%), NDF (neutral detergent fiber, 14.8–25.6%), and condensed tannins (1.74–2.86%). Finally, cyanidin-3-*O*-galactoside was considered an effective compound responsible for antioxidant and anti-glycative activities.

## 1. Introduction

Fruit hulls have been considered the most important bioresources for further processing in food industries, pharmaceutical products, and bioenergy production [1]. In this regard, many fruit hulls have not yet widely been used in the food industry. Pistachio (*Pistacia vera*) is a major nut fruit [2] with a global total annual harvest of close to one million tons. Traditionally, Iran was the largest producer in terms of farmed area, with over 400,000 hectares and of annual harvest and over 300,000 tons of fruit in shell [3]. It was only recently surpassed by the USA and the Republic of Türkiye [4]. In spite of the high biomass of the hulls resulting from shell removal for consumption and processing purposes, they have not yet been applied industrially, and most are disposed of as agricultural waste, while only a small part is applied as animal feed [2]. However, different potentially beneficial properties for the agri-food industry have been reported for pistachio hulls, including antioxidant [5,6], lipid peroxidation and protein degradation [7], increasing biodiesel oxidation stability [2], and improving fish storage [8]. Some preliminary pharmaceutically relevant data indicate cytoprotective activity on lymphocytes, antimutagenicity, and antibacterial activity [6]. Akbari, Kaleghouchi, Ahmadaghaei, and Fandoghi are considered the most important pistachio cultivars in Iran [2]. Interestingly, high variation can be observed between and within the ripe pistachio hulls of different cultivars. For instance, in Kaleghouchi, different hull colors can be observed in full maturity. Thus, the hypothesis can arise that this type of variation could be attributed to different potentially bioactive metabolites in the ripe hulls.

The most important components that lead to different colors are phenolics, such as anthocyanins and other flavonoids [9,10] Based on the previous reports, polyphenolic compounds of pistachio hulls perform important roles in bioactivities such as antioxidant and antimicrobial activity [6]. In addition to polyphenolic compounds, different nutritional parameters of pistachio hulls such as oil, protein, tannin, acid detergent fiber (ADF), and neutral detergent fiber (NDF) should be considered for further food manufacturing [11].

Advanced glycation end products (AGEs) are a heterogenous group of components that are released as a result of a reaction of protein with sugars [12,13,14]. Some oxidative-stress-related diseases, such as diabetes and Alzheimer’s, are associated with accumulation of AGEs [15]. Currently, the use of synthetic drugs for diabetic mellitus is a major concern. The use of plant extracts with high antiglycative and antioxidative effects is of great interest for complementary therapy and prevention. In this regard, the antiglycative activity of pistachio hull extracts in different cultivars can be compared to synthetic ones to introduce new sources of natural antiglycative agents.

*Candida* species are considered a major group of fungal pathogens in humans, especially hospitalized patients. *Candida albicans* inhabits various body surfaces, such as the oral cavity, gastrointestinal tract, vagina, and skin of the healthy individuals as a commensal organism [16]. Moreover, *Candida* species are a common cause of hospital-acquired mycoses. Treating *Candida* infections is regarded as one of the current challenges for medicine. Fluconazole was first introduced as triazole antifungal agent, which has been used as the first-line drug in the treatment of *Candida* infections [17]. Currently, fungal drug resistance caused by the extensive use of antifungal agents has significantly reduced their therapeutic efficacy. Accordingly, there is an urgent need for novel antifungal agents with potent activities and new mechanisms of action to improve the management of *Candida* infections [5]. Moreover, the presence of polyphenolic and anthocyanins in the pistachio hulls as a waste material might provide insights for further introduction of a new source of low-cost anti-*Candida* drugs in the future.

According to a literature survey, there is only one report available regarding polyphenolic compounds of Italian pistachio hull cultivars [7]. However, there is no research on the phenolics and flavonoids in the pistachio hulls of Iranian cultivars, and there is only limited information on some biological activities, such as only one report on antibacterial activity [6]. Moreover, previous reports mostly focused on oil content and fatty acid composition of Iranian pistachio hulls [2], and there is only a single report on the nutritional values of Turkish pistachio hulls. Finally, a comprehensive evaluation of the inter- and intra-cultivar variation of some important Iranian pistachio hull cultivars in respect to their nutritional value, polyphenolic compounds, antiglycative, and antifungal activities has not yet been reported.

The aims of the present research were: (1) to determine the phenolic compounds in the hulls of four major Iranian cultivars, each represented by three genotypes; (2) to evaluate their nutritional values and compare their antioxidant and antiglycative activity based on different food model systems; and (3) to assess the in vitro response of a large collection of *Candida* species to pistachio hull extracts.

## 2. Results and Discussion

### 2.1. Total Phenolic and Flavonoid Content

The total phenolic content (TPC) ranged from 40.6 mg TAE/g DW in AKB2 to 68.1 mg TAE/g DW in KAL1. The results revealed higher TPC in comparison with those obtained [5] in the Fandoghi cultivar with the same unit. However, the use of different solvents can highly affect the amount of TPC [5]. Kazemi et al. [18] also reported 18.8 mg gallic acid/g DW for TPC of pectin obtained from hulls of the Akbari cultivar. A similar trend was also obtained for total flavonols (TFC) (Table 1). The highest and the lowest TFCs were observed in FAN2 (10.93 mg QE/g DW) and AKB3 (4.01 mg QE/g DW), respectively.

### 2.2. Polyphenolic Compounds of Pistachio Hulls

High variation was observed between the studied samples (Table 2). According to HPLC results, cyanidin-3-*O*-galactoside, gallic acid, catechin, and eriodictyol-7-*O*-glucoside were the main components in nine studied pistachio genotypes. Among these genotypes, FAN-2 and KAL-1 possessed the highest contents of cyanidin-3-*O*-galactoside. Bellocco et al. [19] also reported cyanidin-3-*O*-galactoside as the major polyphenolic compound in the ripe pistachio hulls. For catechin content, the FAN1 and AKB1 had the highest (11.01 mg/100 gr DW) and the lowest amounts (7.2 mg/100 gr DW), respectively. For gallic acid, a similar trend was observed.

### 2.3. Antioxidant Activity

In the DPPH radical scavenging model, the highest and the lowest antioxidant activities were obtained in KAL1 and AKB3, respectively (Table 1). A similar trend was also obtained for the ABTS model (Table 1). Ozbek et al. [20] reported high antioxidant activity at 5000 mg/L in the range of 44–97% by β-carotene assay in pistachio hull extracts. It was also determined that the highest antioxidant activity measured using the ORAC test was for 260.9 µmol Trolox equivalents/g extract in 50% ethanolic extract. 

### 2.4. Antiglycative Activity

**Figure 1:**
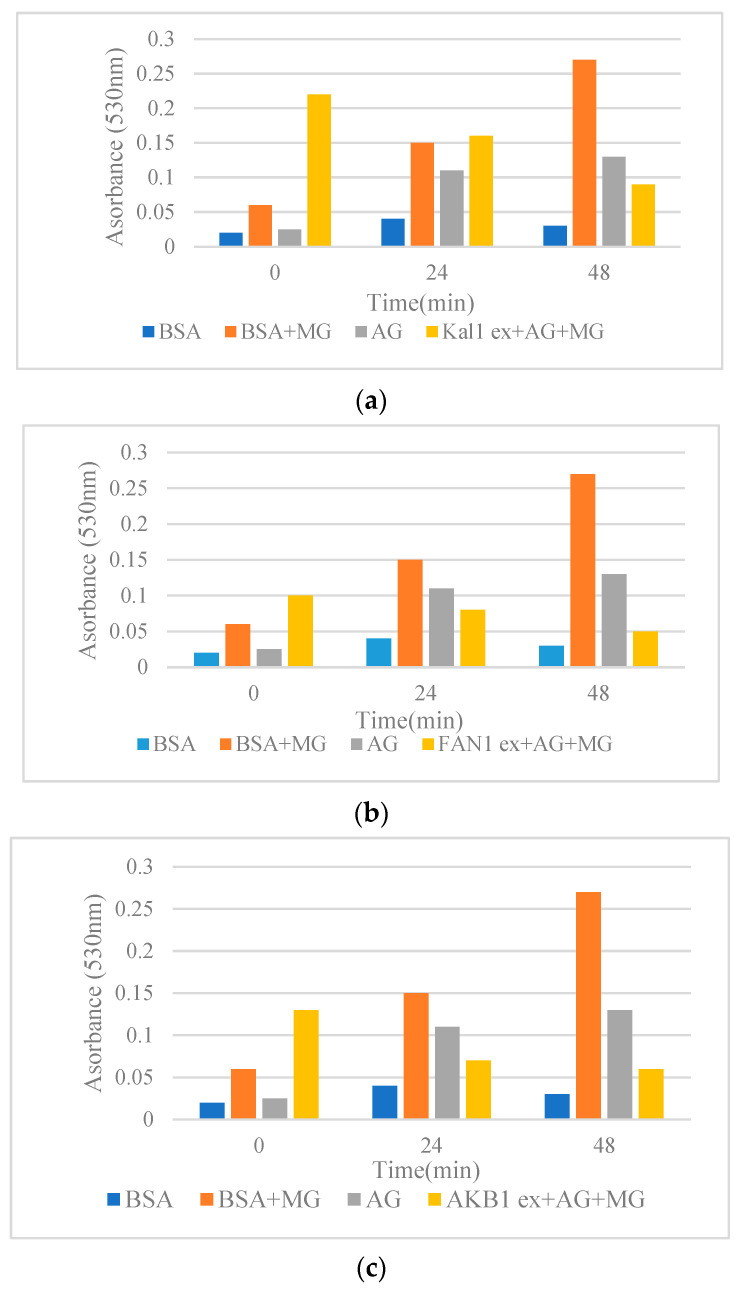

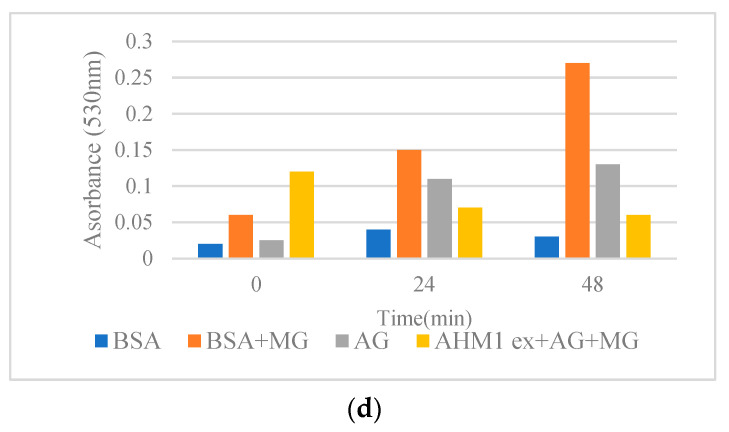
(**a**–**d**) Effects of methanol extracts from pistachio hulls on the cross-β structures in BSA methylglyoxal (MG) model incubated at 50 °C for 48 h. (**a**) Kaleghouchi cultivar, (**b**) Fandoghi cultivar, (**c**) Akbari cultivar, (**d**) Ahmadaghaei cultivar as compared to the positive control, aminoguanidine (AG).

Among the pistachio hull extracts, FAN1 showed higher antiglycative activity (lower absorbance at 530 nm) in comparison with other pistachio extracts (Figure 1). Congo red assay was used to determine the changes in the structure of BSA during glycation. Protein glycation can lead to elevated β-structures formation. In the present research, all the pistachio hull extracts were capable of decreasing the rate of β-structure formation (Figure 1) by inhibiting the transition of α structure to β structure [12]. Data were compared with the positive control, aminoguanidine (AG), known as synthetic anti-glycating agent. Mechanistically, the plant extracts can prevent the modifications in the α-conformers by concealing the glycation sites by decreasing the accessible surface area of the solvent and consequently lead to a reduction in cross-β-structure formation [15]. Previous reports revealed the role of phenolic and flavonoid compounds as the major antiglycative components in *Achillea* species [15], *Foeniculum vulgare* [21], and several Lamiaceae plants [12]. The comparison of antiglycative activity of pistachio hull cultivars with the previous reports using the same method revealed that the absorbance of extracts on the cross-β structures in a BSA methylglyoxal (MG) model at 50 °C after three days for *Satureja* species [12] and *Achillea* [15] was higher than pistachio hull cultivars. This might be due to lower amounts of some phenolic acids in pistachio hulls in comparison with some other medicinal plant leaves. However, as the waste material the antiglycative activity of pistachio hulls might be sufficient for further in vivo research. Previous studies highlighted the role of some polyphenols, such as luteolin caffeic acid and apigenin in *Foeniculum vulgare* [21], and rutin in *Houttuynia cordata* [22]. Moreover, some other researchers attributed the antiglycative properties to methyl or hydroxyl groups in the molecule of a phenolic compound. For example, the effectiveness of chlorogenic acid as an anti-AGE compound is attributed to two additional hydroxyl groups in its cyclohexane and aromatic rings [23].

In this study, cyanidin-3-*O*-galactoside was the major compound. Among the cultivars, Fandoghi genotypes showed higher antiglycative and antioxidant activities. Interestingly, Takabe et al. [24] reported high antiglycative activity of cyanidin-3-*O*-galactoside in *Persicaria hydropiper* sprouts. Cyanidin-3-glucoside and quercetin-3-*O*-galactoside were also major contributors to antiglycative activity in *Vaccinium vitis-idaea* berry [25].

Oxidation performs a critical role in the early steps of glycation; thus, antioxidative capacity might contribute to the antiglycative effect. For these activities, the presence of a hydroxyl group is critical. Cyanidins are widely distributed anthocyanins in fruits and confer a red hue. In most cases, anthocyanins had a higher antioxidant capacity than other flavonoids [26]. Furthermore, anthocyanins such as malvidin, pelargonidin, and peonidin with only one OH group in the B ring showed a lower antioxidant capacity compared to cyanidin with a catechol structure [19,27]. Hence, higher numbers of hydroxyl groups, especially in the B-ring structure of cyanidins, make them potent radical scavengers and consequently antiglycative components [28]. Hodaei et al. [29] also highlighted the role of two hydroxyl groups in the B rings of flavonoids for improvement of the antioxidant capacity in the genus *Chrysanthemum*. Another mechanism that was highlighted by Asgharpour Dil et al. [30] is the reduction of protein cross-linking by plant extracts. Protein cross-linking in extracellular matrix can lead to reduce the flexibility of the proteins, resulting in a thickening of the base membrane, and can increase the damage to organ function, as observed in diabetic nephropathy [30].

### 2.5. Nutrients Analyses

As the pistachio hulls can be used in food industries, their nutritional factors were analyzed. The oil content ranged from 5.1% in FAN2 to 7.6% in AKB1. High variation was also obtained for crude protein. For this element, the highest and the lowest amounts were attributed to FAN1 (10.8%). The ADF content varied from 11.9% in FAN1 to 18.2% in AKB3. A similar trend was also obtained for the NDF content (Table 1). The lowest and the highest condensed tannins were in AHM3 (1.73%) and AKB2 (2.86%), respectively. In a similar study, Boga et al. [11] compared the nutritional values of six Turkish pistachio hull cultivars. The results of the present research were in line with most of their evaluated parameters. Therefore, the pistachio hulls had a moderate level of crude proteins and relatively low levels of tannin and can be suggested for further food products or animal feed.

### 2.6. Antifungal Activity

Table 3 summarizes the results of in vitro antifungal activity of the tested compounds exhibited against the *Candida* species. The AHM2 extract with MIC values of 1.56 and 3.12 µg/mL showed potent activity against *C. albicans*. FAN1, AHM1, AHM2, and AKB1 showed potent activity against *C. glabrata*. Furthermore, the extracts from KAL1, KAL2, FAN1, and AHM1 had a good profile of activity against multi-drug-resistant *C. auris* in comparison with fluconazole. 

The majority of compounds revealed a potent antifungal activity that could be attributed to major polyphenolic or anthocyanin compounds, such as cyanidin-3-*O*-galactoside. However, previous reports highlighted the role of cyanidin derivatives as potent antifungal agents [31,32]. Therefore, in our study, a combination of several components of the polyphenolic profile is probably more important than the amount of any individual compound.

In the present research, pistachio hull cultivars showed different responses to *Candida* species. However, some cultivars revealed potent anti-*Candida* activities. Previous reports revealed that phenolic acids have shown promising in vitro and in vivo activity against *Candida* species [16]. Therefore, the anti-*Candida* activities of pistachio hulls might be attributed to their polyphenolic compounds. Antifungal activities of polyphenolic oligomers may involve interactions with proteins associated with the fungal cell wall [33]. Flavonoids such as quercetin, myricetin, apigenin, and kaempferol have been found to be effective antifungal agents against a wide range of pathogenic organisms [34]. Synergic antifungal properties of quercetin with flucanazole were also reported [34,35]. Similarly, quercetin, resveratrol, and curcumin modulate mitochondrial functions by inhibiting oxidative phosphorylation through various mitochondrial enzymes or by changing ROS generation in mitochondria and by modulating the activity of transcription factors that control mitochondrial proteins’ expression. Naturally occurring flavones, such as apigenin, chrysin, baicalein, luteolin, tangeritin, scutellarein, 6-hydroxyflavone, and wogonin, inhibit efflux pumps, which induces cell death in the fungi. Similarly, flavonols (myricetin, kaempferol, fisetin, quercetin, 3-hydroxy flavone, and 3,7-dihydroxyflavone), a flavone (luteolin), a flavanone (naringenin), and isoflavones (genistein, biochanin A) inhibit the filamentous fungus *Cochliobolus lunatus* through the inhibition of nucleic acid synthesis [35]. Gallic acid extracted from *Paeonia rockii* inhibits the protein synthesis of *C. albicans*, which has been shown to be involved in decreasing the number of hyphal cells and germ tubes with a MIC of 30 mg/mL [36]. Similarly, the synergistic combination with fluconazole inhibits the biofilm of *C. albicans* isolated from patients with vulvovaginal candidiasis. These drugs combined have the ability to avert cell adhesion and cell–cell communication by disturbing the expression of genes accountable for the biofilm formation. These flavonoids are efficient in synergetic combination therapy with conventional drugs, which can be more appropriate and supportive for finding novel drug therapies against fungal pathogens.

### 2.7. Multivariate Analyses

**Figure 2:**
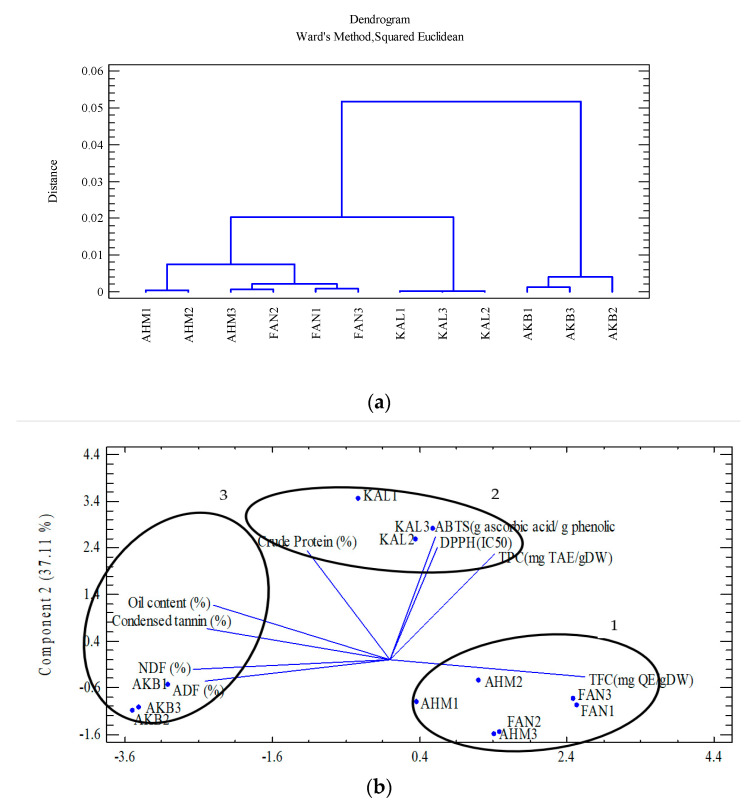
(**a**) Cluster analysis of the studied pistachio cultivars based on polyphenol content, nutrient analysis, and antioxidant activity; (**b**) PCA of the studied pistachio cultivars based on polyphenol content, nutrient analysis, and antioxidant activity.

Cluster analysis was applied to group the 12 studied genotypes based on polyphenolic compounds, nutrients, and antioxidants (Figure 2a). Consequently, the dendrogram classified the genotypes into three groups. Group 1 included Ahmadagaei and Fandoghi cultivars (five genotypes), while Kaleghouchi and Akbari were categorized into Groups 2 and 3, respectively. Principle component analysis (PCA) also confirmed the results obtained via the cluster analysis (Figure 2b). Group 1 is considered a high-flavonoid group (TFC), while Kaleghouchi genotypes (Group 2) were potent in antioxidant capacity. Finally, Akbari genotypes (Group 3) showed a potential for high nutritional values.

## 3. Materials and Methods

### 3.1. Plant Materials

The pistachio hulls were harvested from four cultivars viz. *P. vera*. cv. Akbari, *P. vera*. cv. Kaleghouchi, *P. vera*. cv. Ahmadaghaei, and *P. vera*. cv. Fandoghi in September 2019 from Anar, Kerman (30°52′24″ N and 55°16′14″ E). Each was represented by three genotypes. In each genotype, sampling (1 kg in each sample) was performed in replicate from three trees in the same cultivated field. The soil characteristics of the studied field were EC = 9.56 dSm^−1^. The botanical identification of the collected samples was performed by Dr. Mehdi Rahimmalek based on *Flora Iranica* [37], and the samples were deposited in the herbarium of Isfahan University of Technology. For this purpose, the pistachio fruits were harvested in the afternoon, and the hulls were separated and subjected to shade drying at room temperature (25 °C) over a period of seven days. 

### 3.2. Oil Content

The oil extraction was carried out based on Nouraei et al.’s [38] method. The dried hulls (30 g) were ground into powder using a laboratory mill. The oil was extracted with *n*-hexane. For this purpose, the solvent was mixed with ground pistachio hulls in a 2:1 ratio and stirred for 46 h at 25 °C. The particle size of samples was homogenized through a sieve. Finally, a rotary vacuum evaporator was applied to separate the oil from the solvent. All experiments were repeated in triplicate.

### 3.3. Nutritional Parameters

The Kjeldahl method was applied to measure nitrogen (N) contents as well as to calculate crude protein contents based on the Boga et al. [11]. Van Soest and Wine’s [39] method was used to measure the neutral detergent fiber (NDF) and acid detergent fiber (ADF). 

### 3.4. Condensed Tannins Evaluation

Condensed tannins were also evaluated, as described by Boga et al. [11], in Turkish pistachio hulls using the butanol–HCl method. The insoluble polyvinyl pyrrolidone PVPP–tannin complexes were prepared by suspending the PVPP in aqueous solutions of purified tannins or the tannic acid and stirring the contents for 20 min in the cold.

The contents were stirred for 30 min and centrifuged. The upper phase was used to read the absorbance at 280 nm in comparison with the untreated tannin solution.

### 3.5. Methanolic Extraction

Methanolic extraction was performed for evaluating the phenolics, flavonoids content and antioxidant capacity of the samples. The dried pistachio hulls (2.5 g) were powdered and extracted with methanol (80%) according to the methods described by Tohidi et al. [40]. For this purpose, the samples were placed on an orbital shaker (150 rpm) for 24 h at 25 °C, and the extract was filtered three times.

### 3.6. Total Phenolic and Flavonoid Content

Total phenolic content (TPC) was evaluated based on Folin Ciocalteau’s colorimetric method that was described by Gharibi et al. [41]. The TPC was expressed as milligram of tannic acid equivalent (TAE) per gram of dry weight of the sample. Total flavonoid content (TFC) was determined using the colorimetric aluminum chloride method described by Tohidi et al. [40]. For this purpose, a volume of 125 µL of the pistachio extract was added to 75 µL of a 5% NaNO_2_ solution. The blend was kept for 5 min before 150 µL of AlCl_3_ (10%) was added and incubated for 5 min. Then, 750 µL of NaOH (1 M) was added. The final volume was raised to 2500 µL using distilled water. Finally, the mixture color changed to pink, and the absorbance was evaluated at 510 nm. The total flavonoid content (TFC) was calculated as milligrams of quercetin equivalents (QE) per gram of dry weight of the sample.

### 3.7. Antioxidant Activity

#### 3.7.1. DPPH Assay

The DPPH radical scavenging activity of pistachio hull extracts was performed based on the method reported by Gharibi et al. [41]. Butylatedhydroxytoluene (BHT) was used as the standard synthetic antioxidant. The EC_50_ was evaluated based on plotting the extract concentration versus the corresponding scavenging activity [29].

#### 3.7.2. ABTS Assay

ABTS assay was performed using Barreca et al.’s [7] method. The antioxidant activity was expressed as grams of ascorbic acid per gram of phenolic of the free radical scavenging, compared to the initial. 

### 3.8. Extract Preparation for HPLC

The dried pistachio hulls (20 g) were ground to powder and used for extraction with 80% methanol. The extraction was performed using 500 mL of methanol with 150 rpm shaking for 24 h at 25 °C. Then, the extracts were filtered using 0.45 μm membrane (Millipore, Merck, Germany). Finally, the extracts were kept at 4 °C for further analysis.

### 3.9. HPLC Analysis

The pistachio hull extracts were analyzed using HPLC (model Agilent 1090). All phenolic and flavonoid standards were from Sigma-Aldrich with high purities (≥95% purity). The elution was performed using the Gharibi et al. [42] protocol; 20 μL of the hull extract was injected into the analytical column (250 mm × 4.6 mm (5 μm) Symmetry C18 column (Waters Crop., Milford, MA, USA)) with the matching guard column (10 mm × 4 mm I.D.). The mobile phase was 0.1% formic acid in acetonitrile (flow rate of 0.8 mL min^−1^). Solvents A (0.1% of formic acid aqueous solution) and B (0.1% of formic acid in acetonitrile) were used as the mobile phase with a following gradient elution program: a linear increase from 10% to 26% of B (*v*/*v*) for 40 min, then an increase to 65% solvent B for 70 min and finally to 100% solvent B for 75 min. The detection wavelengths were between 200 and 400 nm. The amount of polyphenolic compounds was calculated according to the peak areas based on respective calibration curves for each standard. The results were reported as mg/100 g of the sample dry weight.

### 3.10. Glycated Albumin Preparation

The antiglycative activity was tested according to the method described by Rahimmalek et al. [12]. Accordingly, bovine serum albumin (Sigma–Aldrich, Cat. No. A7906) (BSA, 5 mg/mL) was incubated with methylglyoxal (MG) (10 mM) in phosphate buffer (0.1 M, pH = 7.4 with sodium azide (0.02%)) both with and without pistachio hull extracts. The solutions were filtered before incubation. BSA and BSA-MG were also applied as controls. Then, all the prepared materials were placed in 50 °C for 48 h and finally kept at 4 °C. 

### 3.11. Protein Glycation and AGEs Formation

The anti-AGEs production properties of pistachio hulls were evaluated using the brown-staining method as described by Rahimmalek et al. [12]. The AGEs content of each sample was measured at 340 nm.

### 3.12. Congo Red Assay

This assay was performed based on the method of Miroliaei et al. [43] using spectrophotometry at 530 nm. The pistachio hull extracts + BSA + MG were used as samples, while BSA + MG and BSA were applied as controls in this procedure.

### 3.13. Testing the Antifungal Activity

Based on the Clinical and Laboratory Standards Institute (CLSI) guidelines [44], the antifungal agents were diluted in an RPMI1640 medium (Sigma Chemical Co., St. Louis, MO, USA) buffered at pH 7.0 with 0.165 M morpholinepropanesulfonic acid (MOPS) (Sigma) with L-glutamine without bicarbonate to produce two times their concentrations and distributed into 96-well microdilution trays with final concentrations of 0.063–64 μg/mL and 0.19 to 200 µg/mL for fluconazole (Pfizer, Groton, CT, USA) and each compound, respectively. Fluconazole and stock solutions of all compounds were prepared in dimethyl sulfoxide (DMSO). The final concentration of DMSO in the test wells was >1%. Briefly, homogeneous suspensions were measured spectrophotometrically at the wavelengths of 530 nm to a percent transmission within the range of 75–77. Therefore, the final densities of the inoculum suspensions of the isolate stock ranged within 1 × 10^3^–3 × 10^3^ CFU/mL, as determined by quantitative colony counts on Sabouraud glucose agar (SGA, Difco). After incubation at 35 °C for 24 h, minimum inhibitory concentration (MIC) values were visually determined. The MIC endpoints were determined with the aid of a reading mirror and defined as the lowest concentration of drug that prevents any recognizable growth causing a significant (≥50%) growth diminution compared to the growth of a drug-free control. MICs were determined after 24 h of incubation at 35 °C. *Candida parapsilosis* (ATCC 22019) and *C. krusei* (ATCC 6258) were included as the quality control isolates for each testing run. 

### 3.14. Statistical Analysis

All experiments were repeated in triplicate. The correlation coefficients were calculated using SPSS (version 16; SPSS Inc., Chicago, IL, USA). Cluster and principal component analyses (PCA) were performed to classify the studied cultivars using Stat Graphics ver. 6.

## 4. Conclusions

A comprehensive and comparative study was carried out on four pistachio cultivars with respect to their major polyphenolic compounds, antioxidant, antiglycative, and antifungal activities in the hull. Nutritional values were also assessed. High variation was found between and within the studied pistachio hull cultivars. Moreover, the results of this research provide new insights into the antidiabetic potential of pistachio hulls. Among the studied cultivars, Fandoghi revealed the highest antiglycative activities; the highest TPC, crude protein, and antioxidant activity was in the Kaleghouchi samples; and the oil content, ADF, NDF, and condensed tannins were superior in Akbari. Moreover, the potent antiglycative activity of the Fandoghi cultivar can introduce this cultivar for further in vivo antidiabetic assays. The results also provided some new information regards anti-*Candida* properties of pistachio hull cultivars for further application as natural drugs. Finally, the diversity and confirmed potential of pistachio hulls as an alternative and valuable source of bioactive components can be beneficial for further pharmaceutical and food products.

## Figures and Tables

**Table 1:** Nutrient, total phenol, flavonoid content and antioxidant capacity of the studied Iranian pistachio hull genotypes.

Genotype	TPC (mg TAE/g DW)	TFC (mg QE/g DW)	Oil Content (%)	Crude Protein (%)	ADF (%)	NDF (%)	Condensed Tannin (%)	DPPH (EC_50_)	ABTS (g Ascorbic Acid/g Phenolic)
AHM1	50.2 ± 0.09 ^i^	8.36 ± 0.03 ^f^	5.5 ± 0.03 ^f^	12.7 ± 0.01 ^e^	15.8 ± 0.01 ^d^	18.8 ± 0.09 ^e^	1.92 ± 0.03 ^f^	382.7 ± 0.27 ^f^	3.1 ± 0.02 ^e^
AHM2	51.8 ± 0.04 ^h^	8.74 ± 0.02 ^e^	5.7 ± 0.05 ^e^	11.3 ± 0.02 ^h^	14.7 ± 0.01 ^g^	17.6 ± 0.07 ^i^	1.83 ± 0.01 ^g^	395.4 ± 0.19 ^d^	3.24 ± 0.03 ^d^
AHM3	53.6 ± 0.02 ^g^	9.45 ± 0.04 ^d^	5.3 ± 0.03 ^g^	10.9 ± 0.01 ^i^	14.9 ± 0..05 ^f^	17.9 ± 0.02 ^h^	1.73 ± 0.04 ^j^	377.8 ± 0.21 ^h^	3.02 ± 0.08 ^f^
FAN1	56.3 ± 0.02 ^e^	10.14 ± 0.02 ^b^	5.8 ± 0.03 ^e^	10.8 ± 0.03 ^i^	11.9 ± 0.02 ^l^	14.8 ± 0.03 ^k^	1.82 ± 0.05 ^h^	375.02 ± 0.11 ^i^	3.31 ± 0.04 ^c^
FAN2	54.1 ± 0.04 ^f^	10.93 ± 0.06 ^a^	5.4 ± 0.02 ^g^	11.7 ± 0.02 ^g^	13.7 ± 0.01 ^i^	18.2 ± 0.06 ^g^	1.96 ± 0.03 ^e^	369.11 ± 0.08 ^k^	3.03 ± 0.01 ^f^
FAN3	57.1 ± 0.03 ^d^	9.96 ± 0.05 ^c^	5.1 ± 0.01 ^h^	9.8 ± 0.04 ^j^	12.8 ± 0.06 ^k^	16.9 ± 0.02 ^j^	1.74 ± 0.06 ^i^	383.26 ± 0.34 ^e^	3.34 ± 0.03 ^c^
KAL1	68.1 ± 0.02 ^a^	7.61 ± 0.01 ^h^	7.2 ± 0.01 ^c^	15.8 ± 0.04 ^a^	15.3 ± 0.03 ^e^	20.2 ± 0.02 ^d^	2.31 ± 0.02 ^d^	405.21 ± 0.22 ^a^	3.96 ± 0.02 ^a^
KAL2	65.8 ± 0.09 ^c^	7.03 ± 0.04 ^i^	6.7 ± 0.04 ^d^	14.4 ± 0.03 ^c^	13.3 ± 0.03 ^j^	17.6 ± 0.04 ^i^	2.44 ± 0.01 ^c^	398.11 ± 0.1 ^c^	3.81 ± 0.04 ^b^
KAL3	67.3 ± 0.1 ^b^	7.81 ± 0.02 ^g^	6.6 ± 0.02 ^d^	14.9 ± 0.02 ^b^	14.1 ± 0.01 ^h^	18.7 ± 0.01 ^f^	2.12 ± 0.01 ^d^	400.06 ± 0.19 ^b^	3.93 ± 0.09 ^a^
AKB1	48.1 ± 0.03 ^j^	4.11 ± 0.03 ^k^	7.6 ± 0.02 ^a^	12.2 ± 0.03 ^f^	16.8 ± 0.02 ^c^	22.8 ± 0.04 ^c^	2.68 ± 0.01 ^b^	381.4 ± 0.29 ^g^	3.13 ± 0.08 ^g^
AKB2	40.6 ± 0.08 ^l^	5.22 ± 0.02 ^j^	7.4 ± 0.03 ^b^	13.1 ± 0.04 ^d^	17.1 ± 0.03 ^b^	23.3 ± 0.01 ^b^	2.86 ± 0.01 ^a^	372.11 ± 0.16 ^j^	3.03 ± 0.07 ^f^
AKB3	46.3 ± 0.1 ^k^	4.01 ± 0.02 ^l^	7.2 ± 0.06 ^c^	12.8 ± 0.02 ^e^	18.2 ± 0.02 ^a^	25.6 ± 0.05 ^a^	2.44 ± 0.02 ^c^	367.33 ± 0.1 ^l^	2.98 ± 0.05 ^e^

Means in each column, means followed by same letter are not significantly different according toLSD’s test at 5% level.

**Table 2:** Polyphenolic compounds content (mg/100 g dried hulls) in pistachio hulls from the studied cultivars based on HPLC analysis.

Compounds	t_R_ (min)	AHM1	AHM2	AHM3	FAN1	FAN2	FAN3	KAL1	KAL2	KAL3	AKB1	AKB2	AKB3
**Gallic acid**	5.8	39.62 ± 0.03	36.24 ± 0.01	39.51 ± 0.03	43.18 ± 0.01	45.25 ± 0.04	41.89 ± 0.01	34.95 ± 0.01	33.23 ± 0.01	36.36 ± 0.02	27.89 ± 0.01	28.34 ± 0.01	28.19 ± 0.01
**Cyanidin-3-*O*-galactoside**	22.8	124.31 ± 0.02	146.26 ± 0.02	128.98 ± 0.01	170.59 ± 0.01	181.94 ± 0.03	173.02 ± 0.06	120.81 ± 0.01	144.96 ± 0.02	125.04 ± 0.01	128.69 ± 0.01	132.06 ± 0.04	140.69 ± 0.03
**Catechin**	28.9	9.31 ± 0.01	8.24 ± 0.01	10.93 ± 0.01	11.01 ± 0.03	10.61 ± 0.01	10.96 ± 0.01	8.57 ± 0.01	7.63 ± 0.01	9.86 ± 0.04	7.2 ± 0.01	9.87 ± 0.01	8.06 ± 0.01
**Epicatechin**	40.5	3.63 ± 0.01	3.04 ± 0.01	3.76 ± 0.01	4.03 ± 0.01	4.62 ± 0.01	4.98 ± 0.01	2.93 ± 0.01	3.04 ± 0.01	2.69 ± 0.01	3.09 ± 0.01	3.85 ± 0.02	3.14 ± 0.01
**Eriodictyol-7-*O*-glucoside**	51.2	13.63 ± 0.04	13.42 ± 0.02	14.98 ± 0.01	15.95 ± 0.01	16.02 ± 0.01	13.72 ± 0.01	13.05 ± 0.01	13 ± 0.01	14.86 ± 0.02	8.16 ± 0.01	8.48 ± 0.01	7.23 ± 0.01
**Naringin**	59.3	2.32 ± 0.01	2.87 ± 0.01	2.24 ± 0.01	3.63 ± 0.01	3.45 ± 0.01	3.16 ± 0.01	2.12 ± 0.01	2.74 ± 0.06	2.09 ± 0.01	2.43 ± 0.01	2.89 ± 0.01	2.65 ± 0.04
**Eriodictyol**	64.2	1.61 ± 0.02	1.98 ± 0.01	1.87 ± 0.02	1.61 ± 0.01	1.72 ± 0.01	1.44 ± 0.01	1.57 ± 0.01	1.93 ± 0.01	1.82 ± 0.01	1.74 ± 0.01	1.81 ± 0.01	1.53 ± 0.01
**Quercetin**	69.3	0.41 ± 0.01	0.36 ± 0.01	0.49 ± 0.01	0.6 ± 0.01	0.62 ± 0.01	0.58 ± 0.03	0.38 ± 0.01	0.33 ± 0.01	0.44 ± 0.03	0.36 ± 0.04	0.42 ± 0.01	0.32 ± 0.01
**Naring enin**	71.6	0.31 ± 0.02	0.28 ± 0.01	0.32 ± 0.01	0.51 ± 0.03	0.46 ± 0.01	0.48 ± 0.02	0.28 ± 0.03	0.26 ± 0.01	0.28 ± 0.01	0.27 ± 0.01	0.29 ± 0.02	0.23 ± 0.01
**Luteolin**	73.4	0.49 ± 0.01	0.45 ± 0.03	0.39 ± 0.01	0.54 ± 0.01	0.6 ± 0.01	0.58 ± 0.01	0.47 ± 0.01	0.41 ± 0.02	0.36 ± 0.01	0.41 ± 0.03	0.38 ± 0.01	0.45 ± 0.02
**Kaempferol**	79.6	0.02 ± 0.01	0.01 ± 0.01	0.01 ± 0.01	0.01 ± 0.01	0.02 ± 0.01	0.01 ± 0.01	0.02 ± 0.01	0.01 ± 0.01	0.01 ± 0.01	0.02 ± 0.01	0.01 ± 0.01	0.01 ± 0.01

Means in each column, means followed by same letter are not significantly different according toLSD’s test at 5% level.

**Table 3:** MIC values (µg/mL) of the studied Iranian pistachio hulls genotypes extracts against *Candida* species. C.a (I): *Candida albicans* (ATCC 10231); C.a (II): *Candida albicans* (FDC 3); C.g (I): *Candida glabrata* (ATCC 15545); C.g (II): *Candida glabrata* (FDC 19); C.p (I): *Candida parapsilosis* (ATCC 90018); C.p (II): *Candida parapsilosis* (FDC 2); C.k (I): *Candida krusei* (ATCC 6258); C.k (II): *Candida Krusei* (FDC 17); C.t: *C. tropicalis*; *C. au*: *Candida auris*.

Compound		C.a (I)	C.a (II)	C.g (I)	C.g (II)	C.p (I)	C.p (II)	C.k (I)	C.k (II)	C.t	C.au
**Fluconazole**		0.25	0.5	2	4	1	0.5	8	>32	1	>64
1	AHM1	6.25	12.5	3.12	1.56	6.25	6.12	12.5	6.25	6.25	12.5
2	AHM2	1.56	3.12	1.56	3.12	6.25	3.12	12.5	6.25	6.25	25
3	AHM3	6.25	3.12	3.12	3.12	3.12	12.5	12.5	12.5	12.5	25
4	FAN1	3.12	12.5	1.56	6.25	6.25	6.25	12.5	12.5	6.25	12.5
5	FAN2	6.25	6.25	6.25	6.25	12.5	12.5	25	25	12.5	25
6	FAN3	12.5	12.5	6.25	6.25	12.5	12.5	25	25	12.5	50
7	KAL1	12.5	6.5	3.12	3.12	12.5	12.5	12.5	6.25	12.5	12.5
8	KAL2	3.12	3.12	6.25	6.25	6.25	6.25	6.25	12.5	6.25	12.5
9	KAL3	6.25	12.5	3.12	3.12	12.5	6.25	12.5	12.5	12.5	25
10	AKB1	6.25	12.5	3.12	1.56	12.5	12.5	12.5	12.5	6.25	25
11	AKB2	12.5	12.5	6.25	3.12	25	6.25	12.5	25	12.5	50
12	AKB3	6.25	6.25	3.12	3.12	12.5	12.5	12.5	12.5	6.25	25

## Data Availability

The data will be available on request.
